# Development of a tissue augmented Bayesian model for expression quantitative trait loci analysis

**DOI:** 10.3934/mbe.2020007

**Published:** 2019-09-26

**Authors:** Yonghua Zhuang, Kristen Wade, Laura M. Saba, Katerina Kechris

**Affiliations:** 1 Department of Biostatistics and Informatics, Colorado School of Public Health, University of Colorado Denver Anschutz Medical Campus, Mail Stop B119, 13001 E. 17th Place, Aurora, 80045, USA; 2 Human Medical Genetics and Genomics Program, School of Medicine, University of Colorado Denver Anschutz Medical Campus, 80045, Aurora, USA; 3 Department of Pharmaceutical Sciences, Skaggs School of Pharmacy and Pharmaceutical Sciences, University of Colorado Denver Anschutz Medical Campus, 80045, Aurora, USA

**Keywords:** eQTL, Bayesian model, allele-specific expression

## Abstract

Expression quantitative trait loci (eQTL) analyses detect genetic variants (SNPs) associated with RNA expression levels of genes. The conventional eQTL analysis is to perform individual tests for each gene-SNP pair using simple linear regression and to perform the test on each tissue separately ignoring the extensive information known about RNA expression in other tissue(s). Although Bayesian models have been recently developed to improve eQTL prediction on multiple tissues, they are often based on uninformative priors or treat all tissues equally. In this study, we develop a novel tissue augmented Bayesian model for eQTL analysis (TA-eQTL), which takes prior eQTL information from a different tissue into account to better predict eQTL for another tissue. We demonstrate that our modified Bayesian model has comparable performance to several existing methods in terms of sensitivity and specificity using allele-specific expression (ASE) as the gold standard. Furthermore, the tissue augmented Bayesian model improves the power and accuracy for local-eQTL prediction especially when the sample size is small. In summary, TA-eQTL’s performance is comparable to existing methods but has additional flexibility to evaluate data from different platforms, can focus prediction on one tissue using only summary statistics from the secondary tissue(s), and provides a closed form solution for estimation.

## 1. Introduction

Understanding the specific biological effect of genomic variants in cells and tissues may provide insight into the mechanisms of disease and complex phenotypes [1]. RNA expression levels of different protein-coding genes may be responsible for mediating the connection between genetic variants and disease susceptibility. Genome-wide association studies (GWAS) have demonstrated that less than 10% of disease-associated genetic variants alter coding sequences. In fact, more than 90% of GWAS-identified genetic variants are located in non-coding regions of the genome (e.g., promoter regions enhancers, non-coding RNA genes), which indicates that these variants might be regulatory [2-4]. The analysis of such genetic variants in the context of gene expression has established an area of genetics focused on identifying expression quantitative trait loci (eQTL) [5]. The Genotype-Tissue Expression (GTEx) project is a realization of this effort [6], and provides a comprehensive public resource of gene expression and genetic data to study tissue-specific gene expression and regulation.

An eQTL is a locus that explains part of the variation in gene expression levels in either inbred populations (e.g., laboratory mice), or outbred populations (e.g., humans) [1, 7]. A standard eQTL study examines the direct association between markers of genetic variation, such as Single Nucleotide Polymorphisms (SNPs), and mRNA expression levels typically measured in tens or hundreds of individuals. This analysis can help reveal biological processes through genetic factors associated with disease [8]. Determining if mRNA expression levels are altered by specific genetic variants provides evidence of a mechanistic link between genetic variation and downstream biological events, of which the first step is often changes in gene expression. SNPs that contribute to these changes in gene expression can be either proximal or distal to the physical location of the gene of interest.

**Figure 1.**
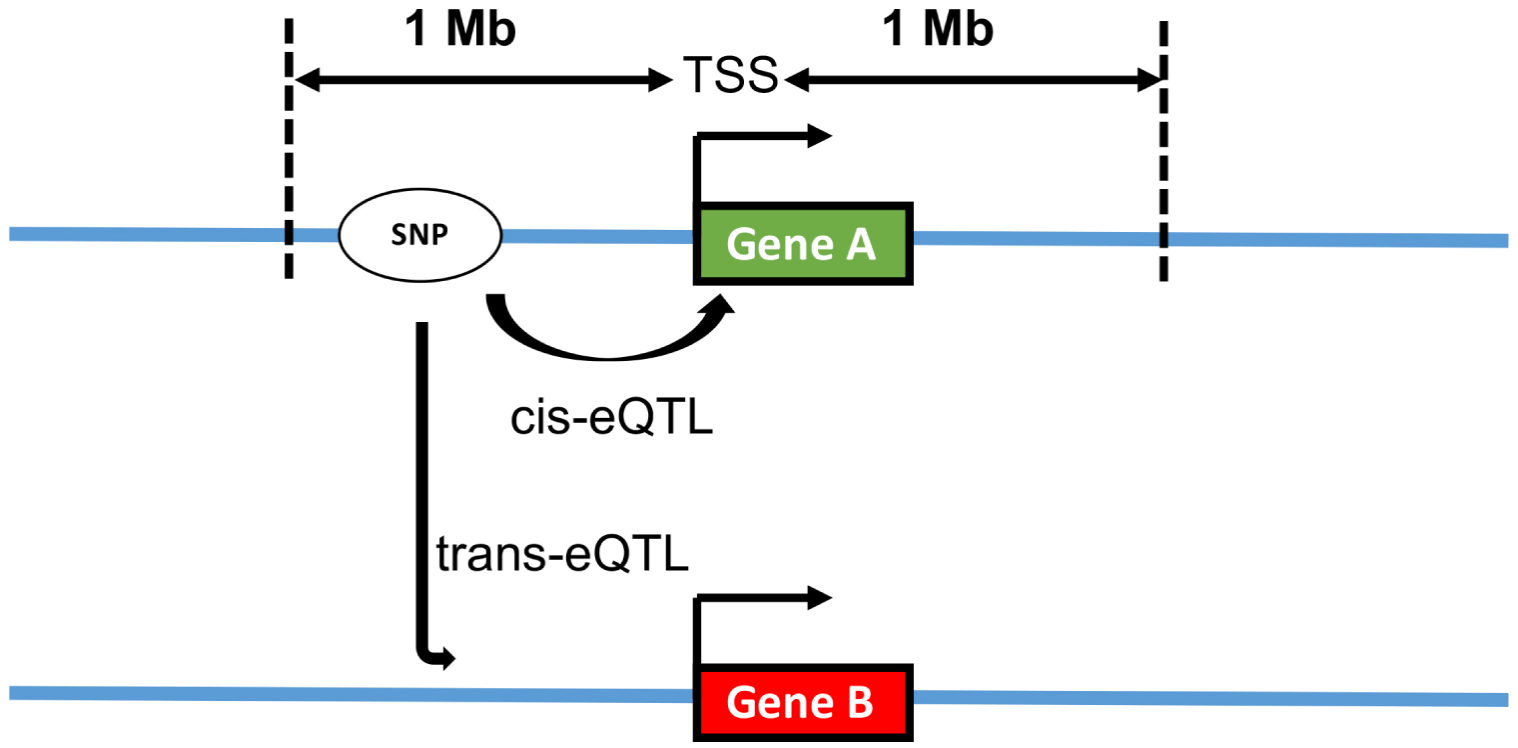
Illustration of cis and trans expression quantitative trait loci (eQTLs). SNP, white circle; gene A, green rectangle (same chromosome); gene B, red rectangle (different chromosome). Each blue line represents different chromosomes.

eQTLs that map to the approximate location of the gene (generally within 1 Mb) are referred to as local-eQTL while those that are far from the location of the gene, often on different chromosomes, are referred to as distal-eQTLs [9]. These two types of eQTLs are sometimes also referred to as *cis* and *trans*, respectively, because local-eQTLs are assumed to act in *cis* and distal-eQTLs are assumed to act in *trans* [10] (Figure 1). In our work, we use the terms local-eQTL and distal-eQTL because we are not able to make conclusions about *cis* or *trans* acting mechanisms respectively without further validation [11]. Several studies suggest that most of the regulatory control takes place locally, *i.e.*, in the vicinity of genes [12-14]. Numerous genes have been detected to have local-eQTLs while detecting distal-eQTLs has been more challenging. Of note, some local-eQTLs are detected in many tissue types while the majority of distal-eQTLs are tissue-dependent [15].

The conventional approach to eQTL analysis is to perform individual tests for each gene-SNP pair using simple linear regression with the number of minor alleles as the independent variable. To choose the most promising SNPs for further evaluation and analysis, the traditional approach simply selects the SNP with the smallest association P-values from standard maximum likelihood tests [16]. This conventional method for eQTL study suffers several limitations. The eQTL analysis with linear regression assumes that every SNP has an equal likelihood of causality and works independently on the targeted gene, which might not be the case. In the conventional study, the large number of genetic markers and expression traits and their complicated correlations lead to a multiple-testing problem [17]. Appropriately making corrections for multiple-testing is a challenge for eQTL studies. In addition, causal SNPs may not exist or be genotyped for some targeted genes. Finally, the conventional eQTL linear regression is performed on each tissue separately and ignores the extensive information known about the SNP effect on RNA expression in other tissue(s), which results in lower power and accuracy due to a limited sample size in the tissue of interest.

To address the problems mentioned above, Bayesian models have been introduced for eQTL, in addition to GWAS, analysis [16, 18-21]. M. Banterle *et al.* recently developed a Bayesian Variable Selection (BVS) model, which allows multiple phenotypes to be associated with multiple genetic predictors (a seemingly unrelated regressions framework) in one tissue [22]. Bayesian models provide a natural modeling framework for eQTL analysis, where information shared across markers and/or genes can increase the power to detect eQTLs [16, 23]. These models are usually based on some modification of a linear model relating expression to SNP genotype(s) [16, 19, 21]. In most cases, non-informative priors are assigned, or hyper-parameters for the priors are set to arbitrary values. To date, many eQTL analyses have studied the association of gene and SNP within a single tissue, but some methods also incorporate multiple tissues [24, 25]. In only a few studies, the informative priors of eQTL include information on that eQTL from a different tissue [26, 27]. Recently, Dr. Li and colleagues developed an empirical Bayes approach for multiple tissue eQTL analysis (MT-eQTL) [27]. Although MT-eQTL accommodates variation in the number of samples, it was not designed to deal with the unequal number of gene transcripts among multiple tissues, resulting from different platforms. Therefore, the MT-eQTL method only performs analyses on the overlapping gene probesets or transcript for eQTL prediction and ignores other transcript information which are not in all tested tissues. This can be problematic if data on different tissues were collected from different array or sequencing platforms.

At the molecular level, comparisons across tissues are often conducted to identify conserved expression changes. For eQTL, we hypothesize that mechanisms for transcriptional control of general processes through SNPs may be conserved across tissues and integrating known eQTL results in one tissue to inform the prediction of eQTL in another tissue will improve power and accuracy. Since eQTL analysis, especially distal-eQTL detection, is a computationally intensive task, we focus on local-eQTL analyses in this study. To improve the accuracy of local-eQTL prediction, we develop a tissue augmented Bayesian model of eQTL (TA-eQTL),which takes prior eQTL information into account to better predict eQTL in another tissue. As an example of TA-eQTL, in a recombinant inbred mouse panel, we incorporate results of lung eQTL to increase power and accuracy of liver eQTL prediction.

The current Bayesian eQTL models were often evaluated based on simulated data [26-28], or on a small number of previously known causal SNPs [16]. Performance assessment on real data is often limited because of an overemphasis on the number of detected eQTLs while ignoring potential false positives. The performance of prediction models can be better assessed using other methods and benchmarks, such as allele-specific expression (ASE). Therefore, in this study we first evaluate performance of methods based on liver ASE-verified local-eQTL data rather than only utilizing simulated data. We also perform sub-sampling to evaluate the benefit of TA-eQTL with decreasing sample size.

## 2. Materials and method

### 2.1. Study subjects: BXD inbred mice

Gene expression data and SNP genotypes in BXD inbred mice were downloaded from the GeneNetwork website [29, 30]. The BXD panel of recombinant inbred (RI) strains were derived by crossing C57BL/6J (B6) and DBA/2J (D2) inbred mouse strains and inbreeding progeny for 20 or more generations. The BXD RI strains has been successfully used to study the genetics of several behavioral phenotypes including alcohol and drug addiction, stress, and locomotor activity [31, 32].

### 2.2. Expression data for BXD

The liver gene expression data for 30 BXD strains of male mice, aged 16 weeks, were generated using the GeneChip Mouse Genome 430A Array and available in NCBI/GEO series GSE16780 [33]. Normalized expression data were downloaded from the GeneNetwork website (GN373). The GeneChip Mouse Genome 430A Array from Affymetrix is a single array representing approximately 14,000 well-characterized mouse genes. The data set includes data from 99 mouse strains, including 30 BXD strains and additional strains in the Hybrid Mouse Diversity Panel [33], with multiple mice per strain profiled. The scanned image data was processed using the Affymetrix GCOS software and the Robust MultiArray method (RMA) to estimate the RNA expression levels of each gene [33].

The lung gene expression data set for 45 BXD strains of male and female mice, aged 49 to 91 days, were generated using the Mouse Genome 430 2.0 Affymetrix array [34]. Normalized expression data were downloaded from GeneNetwork website (GN160). The Affymetrix Mouse Genome 430 2.0 Array offers complete coverage of the Mouse Expression Set 430 and 430A for analysis of over 39,000 transcripts on a single array. The data set includes 45 BXD strains and reciprocal F1 hybrids (B6D2F1 and D2B6F1), and multiple mice per strain were profiled.

For both data sets, GeneNetwork provides the expression of transcripts averaged by strain on a log2 scale, and to simplify comparisons among different data sets, log2 values are adjusted to an average expression of 8 units and a standard deviation of 2 units (variance stabilized). In summary, the 30 BXD strains in the liver gene expression dataset, and the 45 BXD strains in the lung gene expression dataset are used for further analysis.

### 2.3. Genotype data (SNP) for BXD

The BXD genotype data file were downloaded from the GeneNetwork website (http://www.genenetwork.org/genotypes/BXD.geno) on November 30, 2015. A total of 96 BXD strains with 3811 SNPs were obtained. The great majority of SNP genotypes were generated on the Illumina SNP BeadArray. Although there is no distance standard to define local-SNPs, conventionally, variants within 1 Mb (megabase) on either side of a gene’s transcription start site (TSS) are considered local-SNPs while those variants affecting gene expression at a distance greater than 1 Mb from the TSS or on another chromosome are considered distal-SNPs [35, 36].

### 2.4. RNA expression data pre-processing

The gene expression data in mouse liver and lung obtained from Mouse Genome 430A Array (22690 probesets) and 430 V2 Arrays (45119 probesets) were annotated with Ensembl 85: Mus musculus genes (GRCm38.p4) using the Biomart online tool (http://uswest.ensembl.org/biomart/) to retrieve the Ensembl Gene ID and gene location corresponding to the transcript. Of note, we were only able to retrieve Ensembl Gene IDs and gene locations for 20651 probesets (corresponding to 10594 unique genes) and 33684 probesets (corresponding to 14695 unique genes) probe sets in liver and lung expression data, respectively. The shared 10575 unique genes is the set used in further analyses.

### 2.5. SNP data pre-processing

The original SNP data include 3811 markers across 93 BXD strains mice. These SNPs are located on Chromosomes 1-19 and Chromosome X. The SNPs in BXD inbred mice were originally coded as B, D, H (heterozygous) and U (unknown). The heterozygous and unknown SNPs were excluded from analysis due to the uncertainty of their validity. Therefore, we re-coded the SNPs B, D, H ,U, as 0, 1, NA and NA, respectively. The SNP locations were updated to the Ensembl variation 85: Mus musculus genes (GRCm38.p4) version using the Biomart online tool (http://uswest.ensembl.org/biomart/). Among 3811 SNP markers, the chromosome locations were only available for 3023 SNPs in the GRCm38.p4 annotation database.

### 2.6. Allele-specific expression (ASE) in mouse liver

Dr. Lagarrigue *et al.* have analyzed allele-specific expression (ASE) and parent-of-origin expression in adult mouse liver using next generation sequencing (RNA-Seq) in reciprocal crosses of heterozygous F1 mice from the BXD RI parental strains, C57BL/6J and DBA/2J [37]. An exon was considered to have ASE if its adjusted P-value (false discovery rate, FDR) ≤ 0.05 and the expression ratio of the strains (C57BL/6J to DBA/2J) is significantly greater than 1.5 or less than 1/1.5. P-values were calculated using a Fisher’s exact test with the Benjamini-Hochberg method adjustment to control for multiple testing. In this study 397 exons (284 genes)were identified to demonstrate ASE, across three diet contexts, in which 272 ASE genes were found in mice with mouse standard diet (chow). We downloaded all 272 significant ASEs identified in chow-fed mice from the Supplemental Information of [37], and used them as a “gold standard” to evaluate the performance of our newly developed Bayesian methods. Of note, only 192 of 272 ASE genes overlap our shared set of 10575 genes from the gene expression studies. These 192 ASE genes are considered to have true local-eQTL (positive cases) while all others genes (10383) are not considered to have a true local-eQTL (negative cases), and will be used to evaluate performance of different methods.

### 2.7. Tissue augmented Bayesian model of eQTL (TA-eQTL)

Here we describe our method (TA-eQTL), where we extend the basic Bayesian linear regression framework [16, 38] and developed a model that utilizes informative priors based on eQTL results from other tissue(s). In our example, we use evidence of a lung tissue eQTL as a prior for a liver tissue eQTL on a panel of recombinant inbred mice.

#### 2.7.1. Basic local-eQTL model

First, a basic linear model relating lung gene expression to genotype is

(2.1) $$y_{(L)gi}=\alpha_{(L)gk}+\beta_{(L)gk}x_{ki}+\varepsilon_{(L)gki},$$

- *y*(*_L_*)_*gi*_ is the mean expression level of gene *g* = 1, … , *G* in strain *i* = 1, … , *n* in *Lung* tissue;
- *x*_*ki*_ is the genotype for SNP *k* = 1, … , *K* and strain *i* coded as 0 and 1 since all SNPs are homozygous in inbred populations;
- *ε*_(*L*)_*gki*__ is the *Lung* tissue error term for strain *i*, gene *g*, and SNP *k*;
- *α*_(*L*)_*gk*__ is the *Lung* tissue, gene (g), and SNP (*k*) specific intercept;
- *β*_(*L*)_*gk*__ is the *Lung* tissue, gene (*g*), and SNP(*k*) specific coefficient.

The error term is assumed to have a Gaussian distribution, $N(0,\sigma_{(L)gk}^{2})$.

As with the mouse lung eQTL analysis, a similar basic model relating liver gene expression to genotype is

(2.2) $$y_{(V)gi}=\alpha_{(V)gk}+\beta_{(V)gk}x_{ki}+\varepsilon_{(V)gki},$$

- *y*_(*V*)_*gi*__ is the mean expression level of gene *g* in the strain *i* in *Liver* tissue;
- *x*_*ki*_ is the genotype for SNP *k* and strain *i* coded as 0 and 1;
- *ε*_(*V*)_*gki*__ is the *Liver* tissue error term for strain *i*, gene *g*, and SNP *k*;
- *α*_(*V*)_*gk*__ is the *Liver* tissue, gene (*g*), and SNP (*k*) specific intercept;
- *β*_(*V*)_*gk*__ is the *Liver* tissue, gene (*g*), and SNP(*k*) specific coefficient.

The error term is also assumed to have a Gaussian distribution, $N(0,\sigma_{(V)gk}^{2})$. In both the liver and lung BXD studies, the environmental and genetic parameters were tightly controlled. Thus, no additional covariates are included.

For estimation of the parameters of the model, each local-SNP is modeled and regressed separately against each gene for lung and liver separately. For simplicity, we only select the SNP *m* with minimum P-value for each gene (*β*_(*L*)_*gm*__ and *β*_(*V*)_*gm*__) for Bayesian prediction. In other words, each gene has only one local-eQTL for further analysis. The specific SNP selected to represent the local-eQTL for each gene might not be the same between liver and lung tissues. This makes our method more flexible compared to other methods to integrate data from different SNP and gene expression platforms.

- *β*_(*L*)_*gm*__ is the specific coefficient for gene (*g*) and SNP with minimum P-value (*m*) in *Lung* tissue;
- *β*_(*V*)_*gm*__ is the specific coefficient for gene (*g*) and SNP with minimum P-value (*m*) in *Liver* tissue;

The least squares fit is used to obtain estimates of ${\hat{\sigma }}_{(V)gm}^{2}$. Moving forward, we drop the *m* subscript notation for ${\hat{\beta }}_{(V)g}$ and ${\hat{\sigma }}_{(V)g}^{2}$, since only one SNP *m* is considered for each gene.

#### 2.7.2. Prior distribution

We assume the following prior distribution for the coefficient *β*_(*V*)_*g*__ based on the covariate *Z*_(*L*)_ using prior information from lung (*L*).

(2.3) $$\beta_{(V)g}=Z_{(L)g}\Gamma +U_{g},\;\;U_{g}∼N(0,\tau^{2}).$$

We describe the components of this prior model below:

- *β*_(*V*)_*g*__ is a vector of the basic local-eQTL model coefficients (2.2) for the gene (*g*) and SNP pair with minimum P-value in liver (*V*) tissue;

$$\underset{G\times 1}{\beta_{(V)g}}=\left[\begin{array}{c} \beta_{(V)1}\\ \beta_{(V)2}\\ \cdots \\ \beta_{(V)G} \end{array}\right]$$

- *Z*_(*L*)_*g*__ is a matrix including the intercept and prior information from *Lung* for the gene (*g*) and SNP pair with minimum P-value in lung (*L*) tissue;

$$\underset{G\times 2}{Z_{(L)g}}=\left[\begin{array}{cc} 1 & z_{(L)1}\\ 1 & z_{(L)2}\\ \cdots & \cdots \\ 1 & z_{(L)G} \end{array}\right]$$

- Γ is a coefficient vector corresponding to the additive contribution of the prior information to the prior mean;

$$\underset{2\times 1}{\Gamma }=\left[\begin{array}{c} \gamma_{1}\\ \gamma_{2} \end{array}\right]$$

- *U* is the error matrix with zero mean and variance *τ*^2^. We assume a common variance and independence across all genes.

Although, we only include one prior information variable in the *Z* matrix as an example, the model is flexible to include any number of prior information variables. In our example, the prior information variable we considered for inclusion as *z*_(*L*)_*g*__, was the negative logarithm of the P-value (significance of each SNP-gene association) multiplied by the sign of the corresponding *β*_(*V*)_*g*__. In this study, we can ignore the directionality of *β*_(*V*)_*g*__ and *β*_(*L*)_*g*__ since the direction of the effect in mouse lung is not relevant to mouse liver because we do not force the exact same genetic variant to be used in both tissues. We can take the absolute value of *β*_(*V*)_*g*__, but that would violate the Gaussian assumption, therefore we multiply by the sign as an alternative so that the results between lung and liver are in the same direction. In summary, we assume that an increase in the statistical significance level of a mouse lung eQTL should inform the eQTL coefficient *β*_(*V*)_*g*__ in liver.

For estimation of the parameters of this model, *β*_(*V*)_*g*__ is regressed on *Z*_(*L*)_*g*__ across genes to obtain the estimates $\hat{\Gamma }$ and ${\hat{\tau }}^{2}$.

#### 2.7.3. Posterior model and parameter estimation

The Gaussian conjugate prior assumption $\beta_{(V)g}∼N(Z_{(L)g}\Gamma ,\tau^{2})$ leads to a closed form solution for the posterior distribution of *β*_(*V*)_*g*__, which will simplify computation. By completing the square, for gene *g*, the posterior distribution of *β*_(*V*)_*g*__, given the data is [39]

(2.4) β(V)g∣x,y,β(L)g,τ2,σ(V)g2∼N(β~(V)g,S(V)g),∣

The posterior variance term *S*_(*V*)_*g*__ is

(2.5) $$S_{(V)g}^{-1}=(\tau^{2}{)}^{-1}+(\sigma_{(V)g}^{2}{)}^{-1},$$

and estimated by plugging in ${\hat{\tau }}^{2}$ and ${\hat{\sigma }}_{(V)g}^{2}$ described above.

The posterior mean ${\tilde{\beta }}_{(V)g}$ is

(2.6) $${\tilde{\beta }}_{(V)g}=(1-\lambda_{g})Z_{(L)g}\hat{\Gamma }+\lambda_{g}{\hat{\beta }}_{(V)g},$$

which is the weighted average of the maximum likelihood estimate (MLE) ${\hat{\beta }}_{(V)g}$ using the basic model without a prior (first stage) and the estimate of the prior mean $Z_{(L)g}\hat{\Gamma }$ (second stage) [16].

As described previously in [16], the “shrinkage” term *λ_g_* is a function of the two variances, $\sigma_{(V)g}^{2}$ from the basic model (2.2) and *τ*^2^ from the prior in (2.3). *λ_g_* indicates how much the MLE is shrunk towards the prior mean $Z\hat{\Gamma }$. *λ_g_* increases to 1 when *τ*^2^ is large (e.g., less informative prior of mouse lung eQTL) and $\sigma_{(V)g}^{2}$ is small, therefore giving less influence on prior, while *λ_g_* decreases to 0 when *τ*^2^ is small (more informative prior) and $\sigma_{(V)g}^{2}$ is large, thereby giving more influence to the prior. The least square estimates for $\sigma_{(V)g}^{2}$, Γ and *τ*^2^ are substituted into the shrinkage term.

In practice, we found that estimates in the prior model $\left(Z\hat{\Gamma }\right)$ may be underestimating ${\hat{\beta }}_{(V)g}$. Thus, we introduced a constant (*c*) weight to rescale the final estimate ${\tilde{\beta }}_{(V)g}$.

(2.7) $$c=\frac{max({\hat{\beta }}_{(V)g})}{max(Z_{(L)g}\hat{\Gamma })}.$$

The weighted Bayesian posterior mean is now estimated by,

(2.8) $${\tilde{\beta }}_{(V)g}=c(1-\lambda_{g})Z_{(L)g}\hat{\Gamma }+\lambda_{g}{\hat{\beta }}_{(V)g}$$

After calculating the posterior mean $\tilde{\beta }$ and variance $\hat{S}$, we determine the posterior probability of $\tilde{\beta }$ below (or above) 0 depending on the sign of $\tilde{\beta }$, $P(\tilde{\beta }<0|\beta_{(L)g},\tau^{2},\sigma_{(V)g}^{2})$ or $P(\tilde{\beta }>0|\beta_{(L)g},\tau^{2},\sigma_{(V)g}^{2})$ respectively, and multiplied by two using the pnorm() function in R (version 3.5.1). This is similar to the contour probability, which is the Bayesian equivalent to a two-sided test in the frequentist framework, and will be used to rank genes and their corresponding local-SNP [40].

### 2.8. Model performance evaluation

#### 2.8.1. Models evaluation based on ASE

To evaluate the TA-eQTL method, we compared the results with liver local-eQTL benchmarks verified in allele specific expression studies. Allele specific expression (ASE) refers to differences in expression between alternative alleles of a gene. ASE is a complementary approach to eQTL for identifying variants that may regulate expression and therefore, ASEs provide a benchmark for comparison. We used the significant ASEs from [37] as a standard to evaluate the performance of our newly developed Bayesian method. Only the 272 ASE genes from this work are considered to have true liver local-eQTL while all other mouse genes were not considered to have liver local-eQTL. According to the ASE gold standard, we were able to determine the sensitivity and specificity of testing methods, which enables us to derive Receiver operating characteristic (ROC) curves and compare the power and accuracy between Bayesian models and other existing approaches. The area under the ROC curve was computed following the trapezoid rule and the 95% confidence interval (CI) was determined with 2000 stratified bootstrap replicates [41]. The DeLong’s significance test [42] was performed to compare the AUCs of two correlated ROC curves with the “roc.test” function in “pROC 1.13.0” package [41].

#### 2.8.2. Comparison with other methods

We compared the performance of the TA-eQTL method with other existing methods, such as the conventional model (linear regression in liver dataset without lung prior information), meta-analysis approach [43, 44], and an empirical Bayes approach for multiple tissue eQTL analysis (MT-eQTL) [27]. We also performed an eQTL analysis using only the lung expression data (conventional lung) to predict ASE genes.

For meta-analysis, we used the Stouffer test. Stouffer’s method converts one-tailed P-values (*P_i_*) from each of *k* independent tests into standard normal deviates (*Z_i_*) and determines the *Z*_*S*_ score $\left(Z_{S}=\frac{\sum_{i=1}^{k}Z_{i}}{\sqrt{k}}\right)$ to estimate an overall P-value [43]. Stouffer’s method is known as the “inverse normal” or “Z-transform” method [43, 45]. We used a two-sided P-value test for the conventional liver and lung local-QTL analyses (not one-sided P-value). The two-sided test is appropriate because we are not interested in the directionality of *β*.

In the MT-eQTL method, a hierarchical Bayesian model is assumed for *Z_λ_* = {*z*_*λ*1_, *z*_*λ*2_, … , *z*_*λK*_}, which is a vector of Fisher transformation $z=\frac{1}{2}\;\log(\frac{1+r_{\lambda k}}{1-r_{\lambda k}})$ of the correlations *r*_*λk*_ between expression and genotype for a gene-SNP pair *λ* across *k* = 1, …, *K* tissues [27]. It is assumed that $Z_{\lambda }|\mu_{\lambda }∼N_{k}(\mu_{\lambda },\Delta )$ and *μ*_*λ*_ denotes the true effect sizes of the gene-SNP pair *λ* across the *K* tissues. The covariance matrix Δ has diagonal values 1 and its off-diagonal values capture the correlations between tissues. In the MT-eQTL model, *μ_λ_* = Γ*_λ_α_λ_*, where Γ_*λ*_ and *α*_*λ*_ are two random vectors of length *K*. The prior vector Γ_*λ*_ indicates whether there is an eQTL in each of the *k* tissues, and has values of 0 or 1, and *α*_*λ*_ is a effect size vector for the gene-SNP pair *λ*. The marginal posterior probability of having an eQTL in each tissue is *P*(Γ*_λk_* = 1∣*Z_λ_*). After using the Expectation-Maximization algorithm for parameter estimation, the MT analysis reports the marginal probability of not having an eQTL, *P*(Γ*_λk_* = 0∣*Z_λ_*), in each tissue. Smaller values of the marginal probability of not having an eQTL indicate higher likelihood of the gene-SNP pair being an eQTL in the tissue [27]. In our present study, we focused on detecting the local-eQTL at a gene level. Thus, we selected the gene-SNP pair with minimum marginal probability of not having an eQTL on liver tissue at gene level for model performance comparison.

#### 2.8.3. Model evaluation by sub-sampling

We hypothesized that the new augmented Bayesian model improves the power and accuracy for local-eQTL prediction when the sample size is small. When sample size decreases, prior information may increase power to detect true eQTL. To address the effect of sample size in our newly developed TA-eQTL method, we sub-sampled the strains in the liver gene dataset but maintained the prior information from the complete lung eQTL data analysis. The conventional liver gene expression data includes 30 BXD strains and we randomly sub-sampled 10 strains, 15 stains, 20 strains and 25 strains without replacement. For each of these sample sizes (10, 15, 20, 25), six random sub-samples were selected. For each random sub-sampling, we calculated the P-value in the conventional liver local-eQTL analysis, the posterior probability in the TA-eQTL prediction, the marginal P-values in the multiple tissue (MT) analysis, the P-value from the meta-analysis and the P-values in the conventional lung analysis. There is a ROC curve for each of the six random sub-samples, which makes comparison difficult. Therefore, we took the geometric mean values of these P-values or probability values to derive a single ROC curve for each method and compare performance across the sample sizes.

In addition, we calculated the AUC among the five tested methods at each of the 6 random sub-samplings for the four different sample sizes (10, 15, 20, 25), and summarized the AUC by the mean, min and max values. Then, linear mixed models were then used to compare the different eQTL methods. The linear mixed models accounted for random effect of sub-sampling and the correlation of samples. The regressions were performed using the “lmer” function in “lme4” package [46] and the pairwise comparisons between methods were done with the “lsmeans” function in “lsmeans” package [47].

### 2.9. Software details

In this study, unless otherwise specified, all data manipulation and data analyses were performed using RStudio (version 1.0153) [48], R (version 3.5.1) [49] with the following packages: MatrixEQTL (2.2) [50], ggplot2 (3.1.0) [51], fBasics (3042.89) [52], xtable (1.8-3) [53], biomaRt (2.38.0) [54], plyr (1.8.4) [55], data.table (1.12.0) [56], pROC (1.13.0) [41], lme4 (1.1-19) [46] and lsmeans (2.30–0) [47].

## 3. Results

### 3.1. Overlap of lung and liver local-eQTL

We performed local-eQTL analysis on 20651 (corresponding to 10594 unique Ensembl annotated genes) and 33684 (corresponding to 14695 unique Ensembl annotated genes) probe sets with 3023 SNPs for mouse liver and lung, respectively. The shared 10575 genes were found to have cis-SNPs, i.e., there is one or more SNPs within 1 Mb on either side of their transcription start site (TSS). From the potential cis-SNPs for a gene, we selected the SNP with the minimum P-value in each tissue for further analyses. First, we examined whether local genomic control of transcript expression levels is conserved across tissues in mice by comparing the observed overlap of conventionally calculated local-eQTL with the expected overlap between liver and lung. The expected number of shared local-eQTL was calculated under the assumption that the likelihood of a local-eQTL in the two tissues is independent.

**Figure 2.**
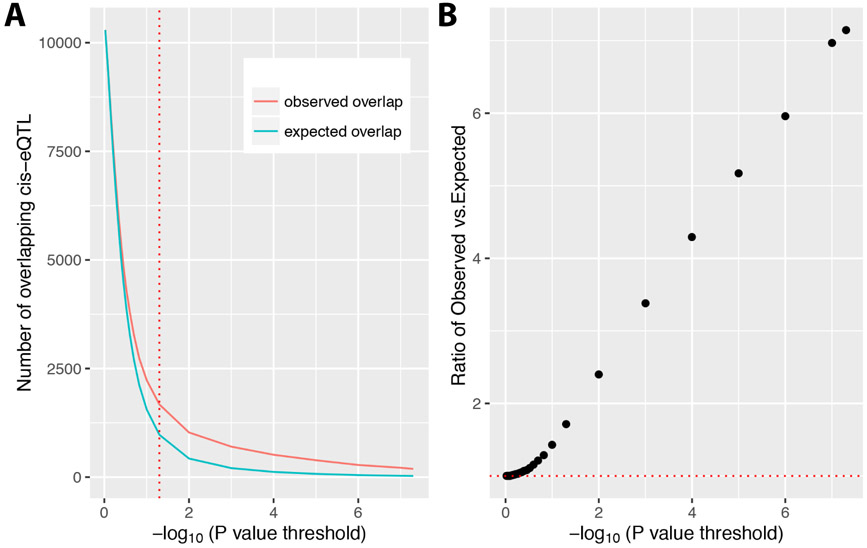
Comparison of overlapping local-eQTL between mouse liver and lung. (A) Number of observed and expected overlapping liver and lung local-eQTL at different local-eQTL significance thresholds. The red dotted line is a nominal threshold of linear regression slope Student’s t-test P-value 0.05 (−*log*_10_(0.05) = 1.3). The blue curve represents the expected number of overlapping local-eQTL, which is calculated under the assumption that the probability of a significant local-eQTLs in mouse liver and lung are independent. The red curve represents the observed number of overlapping local-eQTL. (B) The ratio of the number of observed overlapping local-eQTL to the number of expected overlapping local-eQTL at different significance thresholds. $\text{Ratio}=\frac{Observed\;\;shared\;\;local\;\;eQTL}{Expected\;\;shared\;\;local\;\;eQTL}$. Each black dot represents the ratio between observed and expected local-eQTL in two tissues at different local-eQTL P-value thresholds. The red dotted line represents *ratio* = 1.

We found that the observed number of shared local-eQTL between liver and lung is significantly higher (P-value < 0.05) than the expected overlap at several different P-value thresholds for declaring a local-eQTL significant (Figure 2A). We also observed that the ratio of observed vs. expected $\left(\text{ratio}=\frac{Observed\;\;shared\;\;local\;\;eQTL}{Expected\;\;shared\;\;local\;\;eQTL}\right)$ is positively associated with negative log P-value (Figure 2B). The ratio is 1.71 when the local-eQTL P-value threshold is 0.05. The ratio increases to 9.06 as the local-eQTL P-value threshold becomes more stringent, i.e., negative log P-values increases (Figure 2). We also summarized the number of significant local-eQTL at different P-values thresholds in lung and liver and found that although lung has more, the two sets are fairly similar (Table 1). Consistent with previous work by others, these results suggests that the mechanisms for gene expression control through local SNPs is conserved across tissues, i.e., different tissues share local-eQTL. Thus, it may be useful to take advantage of the known local-eQTL information in one tissue to help predict unknown local-eQTL in another tissue.

### 3.2. TA-eQTL method

**Figure 3.**
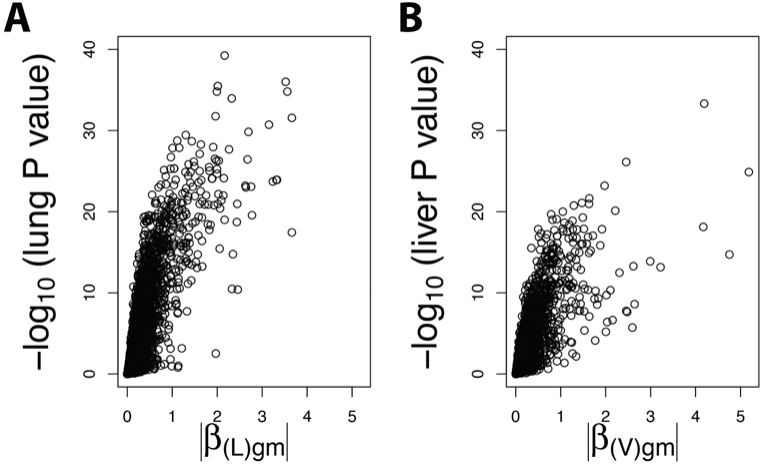
Association between the absolute value of *β* and P-value of local-eQTLs in mouse lung and liver. The *β* values and P-values of local-eQTLs in mouse lung and liver tissues were derived from conventional eQTL analysis (simple linear regression). Gene-SNP pair with minimum P-value at gene level in each tissue is shown. Volcano plots depicts the distributions of absolute *β* values and −*log*_10_(*P*) of local-eQTLs in mouse lung (A) and liver (B).

We developed an augmented Bayesian modeling approach to identify liver local-eQTL using lung local-eQTL results as prior information. Since there is a significant correlation between the *β* magnitude and negative log P-values(*ρ* = 0.84, *P* < 2.2*e* − 16) (Figure 3), we only chose one of them to include as prior information. In this example, we used the negative log of P-values for the lung local-eQTL as a prior for the Bayesian model.

We first used a standard Bayesian model (unweighted) to incorporate lung local-eQTL information to update the liver results. We found that in unweighted Bayesian analysis, posterior estimates $\left(\tilde{\beta }\right)$ were generally lower than the conventional liver prediction $\left(\hat{\beta }\right)$. This is because the prior mean for the local-eQTL $\left(Z\hat{\Gamma }\right)$ are lower than the conventional liver estimates $\left(\hat{\beta }\right)$. The maximum of the prior estimates of the local-eQTL effect in liver, $Z\hat{\Gamma }$, is 0.935, while the maximum of the estimate of the local-eQTL effect in liver derived directly from the liver data, $\hat{\beta }$, is 5.18. To adjust for the distribution difference between $Z\hat{\Gamma }$ and $\hat{\beta }$, we introduced a weight to the Bayesian model. We calculated the weight based on the maximum values of $\hat{\beta }$ and $Z\hat{\Gamma }$, $c=\frac{max(\hat{\beta })}{max(Z\hat{\Gamma })}$. The weighted Bayesian model corrects the imbalance between $Z\hat{\Gamma }$ and $\hat{\beta }$ to create a linear relationship between $\hat{\beta }$ and posterior estimates $\left(\tilde{\beta }\right)$.

In the final step, we calculated the variance of the posterior distribution based on estimates of $\sigma_{(V)g}^{2}$ and *τ*^2^ (Section 2.7). To rank the liver local-eQTL predicted by the weighted Bayesian model, the posterior probability of *β* being less (or greater) than 0 was determined based on the value of $\tilde{\beta }$ and its variance in the normal distribution. We summarized the number of significant local-eQTL at different thresholds for the *β* posterior probability in Table 2.

### 3.3. Model performance assessment

To assess the performance of TA-eQTL, we compared it to liver local-eQTL derived from an allele specific expression (ASE) study [37]. Then we compared the performance of TA-eQTL with several existing methods in terms of sensitivity and specificity using the liver ASE set as the gold standard.

### 3.4. Comparison of TA-eQTL model with ASE local-eQTL

**Figure 4.**
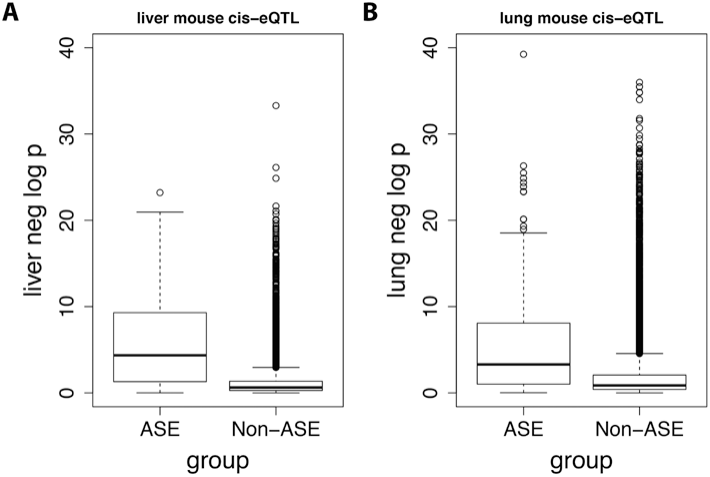
Negative log liver/lung P-value distribution between ASE and Non-ASE groups. Genes were separated into two group, ASE (*n* = 192) and Non-ASE (*n* = 10383) based on the identification of allele specific expression (true cis-eQTL) in liver on chow-fed mice (see Methods). Box plots indicate the distributions of negative log P-value from conventional local-eQTL analyses within the ASE local-eQTL and non-ASE local-eQTL groups in mouse liver (A) and lung (B). The boxes are defined by the top 25th and 75th percentile, and the whiskers are located at 1.5 times the inter-quantile range (IQR).

In the ASE experiment, 272 genes had significant local-eQTL in mice with standard diet (i.e., chowfed). The median of liver negative log P-values is much larger in genes with ASE local-eQTL than the genes without a significant local-eQTL (Figure 4). The trend is maintained when comparing the lung local-eQTL to the ASE local-eQTL from liver, which further suggests that the association between SNP and genes are conserved between liver and lung. Of note, the difference in the median negative log P-values between ASE and Non-ASE groups in mouse lung is less than the difference in liver.

### 3.5. Comparison of TA-eQTL with other methods

**Figure 5.**
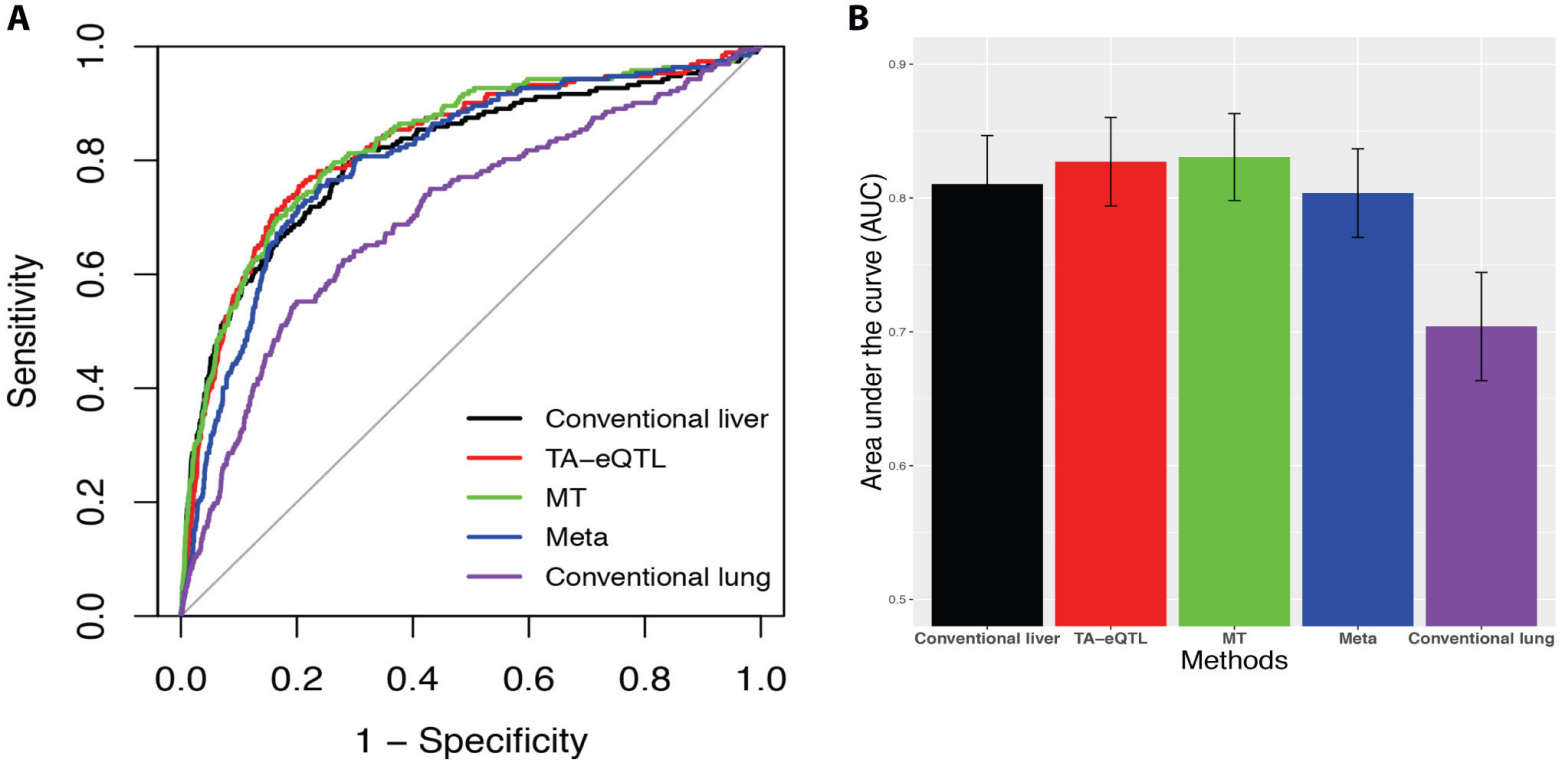
Accuracy comparison of five methods for identifying local-eQTL in liver. (A) The black, red, green, blue and purple lines represent the ROC cures of five analysis methods: conventional liver local-eQTL analysis (no prior), TA-eQTL method, multiple-tissue (MT) Bayesian approach, meta-analysis (meta) and conventional lung local-eQTL analysis. (B) The area under the ROC curves were computed following the trapezoid rule and the 95% confidence interval (CI) was determined through the bootstrap method. Each bar represents the AUC of a prediction method. The error bars represent for the 95% confidence interval. MT and TA-eQTL were significantly different than conventional liver (P-value < 0.05).

P-values or posterior probabilities in each method were used to evaluate each method based on the ASE “gold standard”. Using ROC curves for different P-value/posterior probability cutoffs, we compared the power and accuracy between Bayesian models and other existing approaches (Figure 5). Of note, the closer the ROC curve follows the top-left corner of the ROC space, the more accurate the method. The closer the ROC curve comes to the 45-degree diagonal of the ROC space, the less accurate the method. As shown in Figure 5A, the ROC curve of lung local-eQTL prediction is closest to the 45-degree diagonal, which indicates that it is the least accurate among the five tested methods. Compared with the conventional liver local-eQTL study, the two Bayesian approaches incorporating lung prior knowledge (TA-eQTL method and MT approach) have better performance in predicting liver local-eQTL. The DeLong’s test for ROC curves further reveals that both TA-eQTL and MT methods predict local-eQTL significantly better than the meta analysis, conventional liver analysis and conventional lung analysis (P-value < 0.05). However, the difference between the meta-analysis and the conventional liver study is not significant. In addition, we compared the TA-eQTL and MT ROC curves and their difference is not significant (P-value = 0.60).

To further quantify the performance of the five methods, we calculated the area under the curve (integral) following the trapezoid rule and determined the confidence interval based on a bootstrap strategy. As shown in Figure 5B, the AUCs of the two Bayesian approaches incorporating lung prior knowledge (TA-eQTL method and MT approach) are larger than the AUC of the conventional liver analysis.

### 3.6. Model performance evaluation based on sub-sampling

A major aim in developing the TA-eQTL model is to improve the power and accuracy for local-eQTL prediction when the sample size is small. To address the effect of sample size, we sub-sampled the liver gene dataset but maintained the prior information from the complete lung eQTL data analysis. We compared the area under ROC curves between the TA-eQTL model we developed, and the other 4 approaches under different sub-samplings (25, 20, 15 and 10 strains).

**Figure 6.**
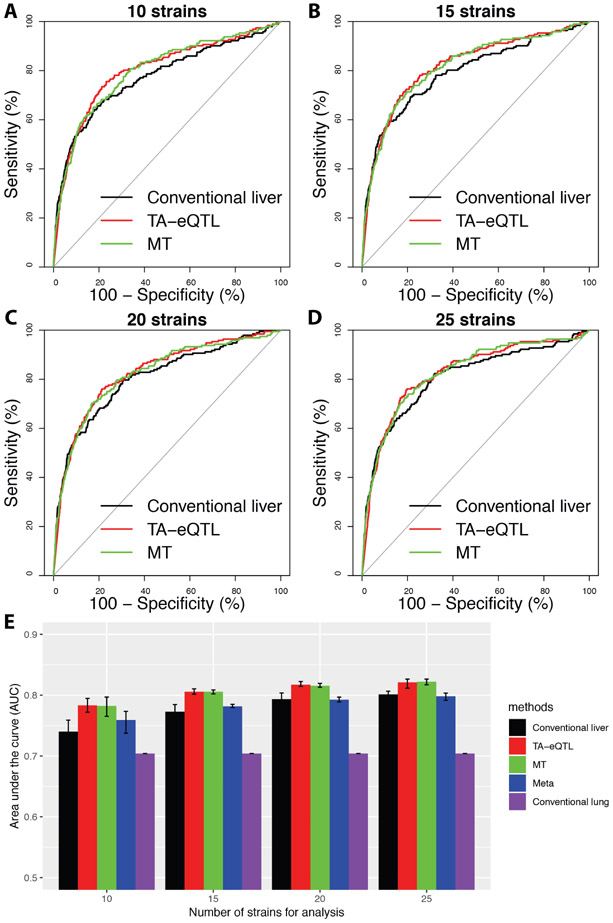
Accuracy comparison of local-eQTL methods across different sample sizes. (A-D) The liver gene expression data were randomly sub-sampled to evaluate the model performances. The sub-sampled liver gene expression data include 10, 15, 20, and 25 strains, respectively. Each sub-sample was randomly performed six times. The black, red and green lines represent the average ROC curves of three methods: conventional liver local-eQTL analysis, TA-eQTL method, and MT approach. (E) Each colored bar chart represents the mean AUC of each prediction method, including conventional lung in purple and Meta in blue. The error bars represents the minimum and maximum values of AUC derived from the six random samples. For 20 and 25 strains, all pairs of methods were significantly different from each other (P-value ≤ 0.001) except for MT and TA-eQTL, and conventional liver and meta (P-value ≥ 0.05). For 15 strains, all pairs of methods were significantly different from each other (P-value ≤ 0.001) except for MT and TA-eQTL (P-value ≥ 0.05). For 10 strains, all pairs of methods were significantly different from each other (P-value ≤ 0.05) except for MT and TA-eQTL (P-value ≥ 0.05).

For the conventional liver local-eQTL analysis with simple linear regression, the AUC decreased quickly when the number of strains decreased and was more sensitive to the number of mouse strains. (Figure 6A-D). For example, the AUC was 0.81 with the full liver dataset (30 strains), but decreased to 0.74 with 10 strains. However, the AUCs of the TA-eQTL method, MT method, and meta-analysis do not decrease as much as the AUC for the conventional liver local-eQTL prediction when sample size decreases (e.g., in TA-eQTL the AUC was 0.83 in 30 strains, and 0.78 in 10 strains). These findings suggest that the three methods incorporating prior information are not as sensitive to the quantity of data as the conventional liver local-eQTL analysis without lung information. Of the three methods that include lung information (TA-eQTL, MT, meta-analysis) the AUC in the TA-eQTL and MT approach are significantly better that the conventional liver for the smallest sample sizes (10, 15) (P-value ≤ 0.001). The two Bayesian methods perform significantly better than meta-analysis in each tested subsampling condition (P-value < 0.001). These results indicate that the TA-eQTL and MT model predicts the liver local-eQTL with higher accuracy than other tested methods, especially when the sample size decreases.

## 4. Discussion

In this study, we developed a tissue augmented Bayesian model of cis-eQTL (TA-eQTL) which was illustrated on the prediction of eQTL in one tissue, by incorporating information from an additional tissue. Although demonstrated on two tissues, our model is also flexible to incorporate any number of tissues, or other covariates as prior information. Bayesian methods provide a natural modeling framework for eQTL analysis to take prior information into account. The prior information shared across tissues can increase the power to detect eQTLs. We focus on the hypothesis that multiple tissue analyses have the potential to improve eQTL predictions [16, 25, 27]. eQTL analyses are generally divided into two categories: gene-level analysis and SNP-level analysis [27]. The former aims at the identification of genes with any local-eQTL while the latter attempt to identify individual SNPs that are significantly associated with a gene. Here we focused on the identification of genes with local-eQTL.

In this study, we first assessed model performance based on liver ASE-verified local-eQTL and compared the newly developed TA-eQTL model and other methods including Multiple Tissue Bayesian method (MT) and meta-analysis. We also evaluated model performance as the sample size decreased. Our results demonstrated that both Bayesian analysis strategies (TA-eQTL and MT) significantly improved local-eQTL gene prediction when compared with the conventional eQTL method and the meta-analysis approach, based on ROC curves and AUC. Although we did not find significant differences between the two Bayesian analysis strategies (TA-eQTL and MT) in the full dataset and sub-sampling analysis, the TA-eQTL method has several advantages. First, TA-eQTL focuses on the prediction of eQTL in a particular tissue, based on prior information from other tissues, while in MT-eQTL, all tissues are treated equally. Second, TA-eQTL is able to summarize a different number of available probesets per gene for the two data sets, which is advantageous when combining existing data from studies that are not perfectly matched by platforms. In these cases, the MT method might not work well since it can only analyze the overlapped probesets across tissues. However, our TA-eQTL method can handle these data since it pre-selects the gene-SNP pair with minimum P-value at the gene level (but not the probeset level) for further Bayesian analysis. Third, TA-eQTL has a closed form solution for posterior estimation, and does not rely on the more computationally intensive iterative EM algorithm for estimation. Four, the TA-eQTL method can be performed using only summary statistics (e.g., P-values) for the secondary tissue(s) in the prior, and therefore does not require individual tissue expression values for the prior. Five, our TA-eQTL is not limited to microarray data. It can also be used on sequencing data since the sequencing count data can be transformed into continuous data using methods such as the variance stabilizing transformation (VST) or Voom [57, 58]. In summary, TA-eQTL provides additional flexibility over the MT-eQTL, while still maintaining similar accuracy performance.

Despite many advantages, there are some limitations of this method. One limitation is that the TA-eQTL method does not efficiently use all of the information contained in these large and complex data sets, by summarizing information at the gene level. For example, one gene could have several significant gene-SNP pairs. Another limitation of this particular study is that the ASE gene list we used as the gold standard to evaluate model performance is not complete because it only captures genes with true cis-eQTL that also have a genetic variant within the transcribed region. In addition, Lagarrigue *et al.* used the Fisher’s exact test for ASE detection, but this approach does not account for over-dispersion in these types of data sets. A beta-binomial distribution framework developed may be more appropriate to model ASE [59]. Both the TA-eQTL and MT methods model individual SNPs and do not consider the potential for gene expression associations with multiple SNPs. Banterle *et al.* have recently developed the Bayesian seemingly unrelated regression (BayesSUR) method, which considers simultaneously many predictors and several outcomes or responses and allows for variable selection and dependence structure between the response variables [22]. In our context, BayesSUR could simultaneously model the association between many SNPs and the expression of multiple genes and account for interdependencies among the SNPs and among genes. This type of multi-dimensional model is a promising direction for the identification of eQTLs. However, for our motivating multiple tissue eQTL study, it is not straightforward to adapt BayesSUR because of the additional layer of multiple tissues in the model. In addition, the BayesSUR approach implements the evolutionary stochastic search MCMC algorithm (ESS), which is more efficient than the Shotgun Stochastic Search algorithm (SSS), but still computationally intensive for high dimensional data [60]. Our method also identifies local-eQTL that may not represent differences in expression due to one allele either being repressed or activated. Like all methods, the occurrence of SNPs in a probeset or gene need to be considered because these may show an artificial difference in expression [61]. Filtering these occurrences in advance or post-hoc are common strategies that can be implemented.

## 5. Conclusion

We presented a Bayesian method, called TA-eQTL, for incorporating prior information in eQTL analysis. As an application, we tested the method in a panel of recombinant inbred mice for the prediction of liver local-eQTL using lung local-eQTL information as a prior. Performance for eQTL prediction is often based on simulations or counting the number of predictions, but not accounting for false discoveries. In this work, we examined performance using allele-specific expression (ASE) as a benchmark. We also evaluated the performance of different methods as sample size decreased. In summary, methods that incorporate information from the lung tissue were better at predicting ASE genes. Our method TA-eQTL and another Bayesian method, MT-eQTL, performed similarly in terms of AUC among the methods especially with decreasing sample size. Although these two methods performed comparably, TA-eQTL is more flexible in that it can handle datasets that are derived from different platforms, in addition to other types of covariates. TA-eQTL also can prioritize one tissue over other tissue(s) in the regression model. Finally, TA-eQTL is computationally tractable for the large number of genes and SNPs that need to be evaluated, since it provides a closed form solution for estimation for each model fit.

## Figures and Tables

**Table 1.** Summary of genes with a significant local-eQTL in each tissue with different local-eQTL P-value threshold using the conventional method.

P value threshold	No. of genes with a significant cis-eQTL in lung (% of total)	No. of genes with a significant cis-eQTL in liver (% of total)
0.05	3609 (34)	2858 (27)
0.01	2477 (23)	1828 (17)
0.001	1774 (17)	1238 (12)
1e-04	1381 (13)	919 (9)
1e-05	1124 (11)	708 (7)
1e-06	922 (9)	539 (5)
1e-07	777 (7)	422 (4)
1e-08	665 (6)	315 (3)
1e-09	550 (5)	245 (2)

**Table 2.** Summary of genes with a significant local-eQTL based on posterior probability.

Posterior probability threshold	No. of genes with significant cis-eQTL in liver (% of total)
0.05	3609 (34)
0.01	2523 (24)
0.001	1768 (17)
1e-04	1410 (13)
1e-05	1202 (11)
1e-06	1035 (10)
1e-07	913 (9)
1e-08	824 (8)
1e-09	739 (7)

## References

[1] Nica AC and Dermitzakis ET, Expression quantitative trait loci: present and future, Philos. Trans. R. Soc. Lond. B Biol. Sci, 368 (2013), 20120362. 23650636 10.1098/rstb.2012.0362 PMC3682727

[2] Hindorff LA, Sethupathy P, Junkins HA, , Potential etiologic and functional implications of genome-wide association loci for human diseases and traits, Proc. Natl. Acad. Sci. U S A, 106 (2009), 9362–9367. 19474294 10.1073/pnas.0903103106 PMC2687147

[3] Hrdlickova B, de Almeida RC, Borek Z, , Genetic variation in the non-coding genome: Involvement of micro-rnas and long non-coding rnas in disease, Biochim. Biophys. Acta, 1842 (2014), 1910–1922. 24667321 10.1016/j.bbadis.2014.03.011

[4] Ricaño-Ponce I and Wijmenga C, Mapping of immune-mediated disease genes, Annu. Rev. Genomics Hum. Genet, 14 (2013), 325–353. 23834318 10.1146/annurev-genom-091212-153450

[5] Jansen RC and Nap JP, Genetical genomics: the added value from segregation, Trends Genet., 17 (2001), 388–391. 11418218 10.1016/s0168-9525(01)02310-1

[6] Carithers LJ, Ardlie K, Barcus M, , A novel approach to high-quality postmortem tissue procurement: The gtex project, Biopreserv. Biobank, 13 (2015), 311–319, 26484571 10.1089/bio.2015.0032 PMC4675181

[7] Cookson W, Liang L, Abecasis G, , Mapping complex disease traits with global gene expression, Nat. Rev. Genet, 10 (2009), 184–194. 19223927 10.1038/nrg2537 PMC4550035

[8] Nica AC and Dermitzakis ET, Using gene expression to investigate the genetic basis of complex disorders, Hum. Mol. Genet, 17 (2008), R129–134. 18852201 10.1093/hmg/ddn285 PMC2570059

[9] Rockman MV and Kruglyak L, Genetics of global gene expression, Nat. Rev. Genet, 7 (2006), 862–872. 17047685 10.1038/nrg1964

[10] Cubillos FA, Coustham V and Loudet O, Lessons from eqtl mapping studies: non-coding regions and their role behind natural phenotypic variation in plants, Curr. Opin. Plant. Biol, 15 (2012), 192–198. 22265229 10.1016/j.pbi.2012.01.005

[11] Fraser HB, Moses AM and Schadt EE, Evidence for widespread adaptive evolution of gene expression in budding yeast, Proc. Natl. Acad. Sci. U S A, 107 (2010), 2977–2982. 20133628 10.1073/pnas.0912245107 PMC2840270

[12] Dixon AL, Liang L, Moffatt MF, , A genome-wide association study of global gene expression, Nat. Genet, 39 (2007), 1202–1207. 17873877 10.1038/ng2109

[13] Göring HHH, Curran JE, Johnson MP, , Discovery of expression qtls using large-scale transcriptional profiling in human lymphocytes, Nat. Genet, 39 (2007), 1208–1216. 17873875 10.1038/ng2119

[14] Schadt EE, Molony C, Chudin E, , Mapping the genetic architecture of gene expression in human liver, PLoS Biol., 6 (2008), e107. 18462017 10.1371/journal.pbio.0060107 PMC2365981

[15] Gerrits A, Li Y, Tesson BM, , Expression quantitative trait loci are highly sensitive to cellular differentiation state, PLoS Genet., 5 (2009), e1000692. 19834560 10.1371/journal.pgen.1000692 PMC2757904

[16] Chen GK and Witte JS, Enriching the analysis of genomewide association studies with hierarchical modeling, Am. J. Hum. Genet, 81 (2007), 397–404. 17668389 10.1086/519794 PMC1950795

[17] Zhang X, Huang S, Sun W, , Rapid and robust resampling-based multiple-testing correction with application in a genome-wide expression quantitative trait loci study, Genetics, 190 (2012), 1511–1520. 22298711 10.1534/genetics.111.137737 PMC3316660

[18] Scott-Boyer MP, Imholte GC, Tayeb A, , An integrated hierarchical bayesian model for multivariate eqtl mapping, Stat. Appl. Genet. Mol. Biol, 11 (2012), 10.1515/1544-6115.1760. PMC4627701 22850063

[19] Stegle O, Parts L, Durbin R, , A bayesian framework to account for complex non-genetic factors in gene expression levels greatly increases power in eqtl studies, PLoS Comput. Biol, 6 (2010), e1000770. 20463871 10.1371/journal.pcbi.1000770 PMC2865505

[20] Stephens M and Balding DJ, Bayesian statistical methods for genetic association studies, Nat. Rev. Genet, 10 (2009), 681–690. 19763151 10.1038/nrg2615

[21] Veyrieras J-B, Kudaravalli S, Kim SY, , High-resolution mapping of expression-qtls yields insight into human gene regulation, PLoS Genet., 4 (2008), e1000214. 18846210 10.1371/journal.pgen.1000214 PMC2556086

[22] Banterle M, Bottolo L, Richardson S, , Sparse variable and covariance selection for high-dimensional seemingly unrelated bayesian regression, bioRxiv, 467019.

[23] Imholte GC, Scott-Boyer M-P, Labbe A, , ibmq: a r/bioconductor package for integrated bayesian modeling of eqtl data, Bioinformatics, 29 (2013), 2797–2798. 23958729 10.1093/bioinformatics/btt485 PMC3799478

[24] Duong D, Gai L, Snir S, , Applying meta-analysis to genotype-tissue expression data from multiple tissues to identify eqtls and increase the number of egenes, Bioinformatics, 33 (2017), i67–i74. 28881962 10.1093/bioinformatics/btx227 PMC5870567

[25] Sul JH, Han B, Ye C, , Effectively identifying eqtls from multiple tissues by combining mixed model and meta-analytic approaches, PLoS Genet., 9 (2013), e1003491. 23785294 10.1371/journal.pgen.1003491 PMC3681686

[26] Flutre T, Wen X, Pritchard J, , A statistical framework for joint eqtl analysis in multiple tissues, PLoS Genet., 9 (2013), e1003486. 23671422 10.1371/journal.pgen.1003486 PMC3649995

[27] Li G, Shabalin AA, Rusyn I, , An empirical bayes approach for multiple tissue eqtl analysis, Biostatistics, 19 (2018), 391–406. 29029013 10.1093/biostatistics/kxx048 PMC6366007

[28] Das A, Morley M, Moravec CS, , Bayesian integration of genetics and epigenetics detects causal regulatory snps underlying expression variability, Nat. Commun, 6 (2015), 8555. 26456756 10.1038/ncomms9555 PMC4633824

[29] Chesler EJ, Lu L, Wang J, , Webqtl: rapid exploratory analysis of gene expression and genetic networks for brain and behavior, Nat. Neurosci, 7 (2004), 485–486. 15114364 10.1038/nn0504-485

[30] Wang J, Williams RW and Manly KF, Webqtl: web-based complex trait analysis, Neuroinformatics, 1 (2003), 299–308. 15043217 10.1385/NI:1:4:299

[31] Phillips TJ, Huson M, Gwiazdon C, , Effects of acute and repeated ethanol exposures on the locomotor activity of bxd recombinant inbred mice, Alcohol. Clin. Exp. Res, 19 (1995), 269–278. 7625557 10.1111/j.1530-0277.1995.tb01502.x

[32] Tabakoff B, Saba L, Kechris K, , The genomic determinants of alcohol preference in mice, Mamm. Genome, 19 (2008), 352–365. 18563486 10.1007/s00335-008-9115-z PMC2583933

[33] Bennett BJ, Farber CR, Orozco L, , A high-resolution association mapping panel for the dissection of complex traits in mice, Genome. Res, 20 (2010), 281–290. 20054062 10.1101/gr.099234.109 PMC2813484

[34] Alberts R, Lu L, Williams RW, , Genome-wide analysis of the mouse lung transcriptome reveals novel molecular gene interaction networks and cell-specific expression signatures, Respir. Res, 12 (2011), 61. 21535883 10.1186/1465-9921-12-61 PMC3105947

[35] Blauwendraat C, Francescatto M, Gibbs JR, , Comprehensive promoter level expression quantitative trait loci analysis of the human frontal lobe, Genome Med., 8 (2016), 65. 27287230 10.1186/s13073-016-0320-1 PMC4903003

[36] Webster JA, Gibbs JR, Clarke J, , Genetic control of human brain transcript expression in alzheimer disease, Am. J. Hum. Genet, 84 (2009), 445–458. 19361613 10.1016/j.ajhg.2009.03.011 PMC2667989

[37] Lagarrigue S, Martin L, Hormozdiari F, , Analysis of allele-specific expression in mouse liver by rna-seq: a comparison with cis-eqtl identified using genetic linkage, Genetics, 195 (2013), 1157–1166. 24026101 10.1534/genetics.113.153882 PMC3813844

[38] Gelman A, Carlin JB, Stern HS, , Bayesian data analysis, vol. 2, Chapman & Hall/CRC Boca Raton, FL, USA, 2014.

[39] Hoff PD, A first course in Bayesian statistical methods, vol. 580, Springer, 2009.

[40] Lesaffre E and Lawson AB, Bayesian biostatistics, John Wiley & Sons, 2012.

[41] Robin X, Turck N, Hainard A, , proc: an open-source package for r and s+ to analyze and compare roc curves, BMC Bioinformatics, 12 (2011), 77. 21414208 10.1186/1471-2105-12-77 PMC3068975

[42] DeLong ER, DeLong DM and Clarke-Pearson DL, Comparing the areas under two or more correlated receiver operating characteristic curves: a nonparametric approach, Biometrics, 44 (1988), 837–845. 3203132

[43] Stouffer SA, Suchman EA, DeVinney LC, , The american soldier: Adjustment during army life, Princeton University Press, Vol. 1.

[44] T. L, On the combination of independent tests, Magyar Tud Akad Mat Kutato Int Közl.

[45] Whitlock MC, Combining probability from independent tests: the weighted z-method is superior to fisher’s approach, J. Evol. Biol, 18 (2005), 1368–1373. 16135132 10.1111/j.1420-9101.2005.00917.x

[46] Bates D, Mächler M, Bolker B, , Fitting linear mixed-effects models using lme4, J. Stat. Software, 67 (2015), 1–48.

[47] Lenth RV, Least-squares means: The R package lsmeans, J. Stat. Software, 69 (2016), 1–33.

[48] RStudio Team, RStudio: Integrated Development Environment for R, RStudio, Inc., Boston, MA, 2015.

[49] R Core Team, R: A Language and Environment for Statistical Computing, R Foundation for Statistical Computing, Vienna, Austria, 2015.

[50] Shabalin AA, Matrix eqtl: ultra fast eqtl analysis via large matrix operations, Bioinformatics, 28 (2012), 1353–1358. 22492648 10.1093/bioinformatics/bts163 PMC3348564

[51] Wickham H, ggplot2: Elegant Graphics for Data Analysis, Springer-Verlag New York, 2009.

[52] Team RC, Wuertz D, Setz T, , fBasics: Rmetrics - Markets and Basic Statistics, 2014, R package version 3011.87.

[53] Dahl DB, xtable: Export Tables to LaTeX or HTML, 2016, R package version 1.8-2.

[54] Durinck S, Moreau Y, Kasprzyk A, , Biomart and bioconductor: a powerful link between biological databases and microarray data analysis, Bioinformatics, 21 (2005), 3439–3440. 16082012 10.1093/bioinformatics/bti525

[55] Wickham H, The split-apply-combine strategy for data analysis, J. Stat. Software, 40 (2011), 1–29.

[56] Dowle M, Srinivasan A, Short T, , data.table: Extension of Data.frame, 2015, R package version 1.9.6.

[57] Anders S and Huber W, Differential expression analysis for sequence count data, Genome biol., 11 (2010), R106. 20979621 10.1186/gb-2010-11-10-r106 PMC3218662

[58] Law CW, Chen Y, Shi W, , voom: Precision weights unlock linear model analysis tools for rna-seq read counts, Genome Biol., 15 (2014), R29. 24485249 10.1186/gb-2014-15-2-r29 PMC4053721

[59] Sun W, A statistical framework for eqtl mapping using rna-seq data, Biometrics, 68 (2012), 1–11. 21838806 10.1111/j.1541-0420.2011.01654.x PMC3218220

[60] Bottolo L and Richardson S, Evolutionary stochastic search for bayesian model exploration, Bayesian Anal., 5 (2010), 583–618.

[61] Walter NA, McWeeney SK, Peters ST, , Snps matter: impact on detection of differential expression, Nature Methods, 4 (2007), 679. 17762873 10.1038/nmeth0907-679 PMC3410665

